# Single *R* Gene Introgression Lines for Accurate Dissection of the *Brassica* - *Leptosphaeria* Pathosystem

**DOI:** 10.3389/fpls.2016.01771

**Published:** 2016-11-28

**Authors:** Nicholas J. Larkan, Fengqun Yu, Derek J. Lydiate, S. Roger Rimmer, M. Hossein Borhan

**Affiliations:** ^1^Saskatoon Research Centre, Agriculture and Agri-Food CanadaSaskatoon, SK, Canada; ^2^Armatus Genetics Inc.Saskatoon, SK, Canada

**Keywords:** *Brassica napus*, *Leptosphaeria maculans*, introgression lines, resistance genes, blackleg, differentical lines

## Abstract

Seven blackleg resistance (*R*) genes (*Rlm1, Rlm2, Rlm3, Rlm4, LepR1, LepR2* & *LepR3*) were each introgressed into a common susceptible *B. napus* doubled-haploid (DH) line through reciprocal back-crossing, producing single-*R* gene introgression lines (ILs) for use in the pathological and molecular study of *Brassica*—*Leptosphaeria* interactions. The genomic positions of the *R* genes were defined through molecular mapping and analysis with transgenic *L. maculans* isolates was used to confirm the identity of the introgressed genes where possible. Using *L. maculans* isolates of contrasting avirulence gene (*Avr*) profiles, we preformed extensive differential pathology for phenotypic comparison of the ILs to other *B. napus* varieties, demonstrating the ILs can provide for the accurate assessment of *Avr*-*R* gene interactions by avoiding non-*Avr* dependant alterations to resistance responses which can occur in some commonly used *B. napus* varieties. Whole-genome SNP-based assessment allowed us to define the donor parent introgressions in each IL and provide a strong basis for comparative molecular dissection of the pathosystem.

## Introduction

In a plant-pathogen system that follow gene-for-gene interactions (Flor, 1971), genetically well-defined resistant host germplasm (differential lines) are an essential tool for identifying and characterizing avirulence (*Avr*) alleles present in pathogen isolates. Ideally, each plant differential line should represent a single resistance (*R*) gene in an otherwise susceptible host genotype, providing a common background for individual *R* genes. In addition to being a valuable tool for pathology, producing single *R* gene differential lines in a common genetic background would allow researchers to dissect and compare the robustness, efficiency and downstream molecular defense pathways for each *R* gene, free from the interference caused by variation in background genotypic effects.

Resistance against the hemibiotrophic fungal pathogen *Leptosphaeria maculans*, the causative agent of blackleg disease in of Brassica species (Howlett et al., 2001), is governed largely by race-specific *R* genes. Nineteen *R* genes, identified in several Brassica species, have been reported to convey race-specific resistance against *L. maculans* (reviewed in Raman et al., 2013). Twelve of these *R* genes have been positioned in the A genome of *B. napus* or *B. rapa* (*Rlm1, Rlm2, Rlm3, Rlm4, Rlm7, Rlm9, LepR1, LepR2, LepR3, LepR4*) or the B genome of *B. juncea* (*LmJR1, LmJR2*) via linkage mapping (Delourme et al., 2004; Yu et al., 2005, 2007, 2012; Christianson et al., 2006; Long et al., 2011; Raman et al., 2012a,b; Larkan et al., 2013, 2014, 2016), while two more have been assigned to B genome chromosomes of *B. juncea* and/or *B. nigra* (*Rlm6, Rlm10*) through marker-based assessment of recombinant or chromosome addition lines (Chèvre et al., 1996, 1997; Eber et al., 2011). *RlmS*, identified in the *B. napus* variety Surpass 400 (Van De Wouw et al., 2009) likely corresponds to the *BlmR2* locus mapped to chromosome A10 (Long et al., 2011; Larkan et al., 2013). The remaining *R* genes (*Rlm5, Rlm8, Rlm11*, and *RlmJ1*) have yet to be genetically characterized in the host Brassica lines in which they reside. However, *Rlm8* and *Rlm11* are contained within the A genome of *B. rapa* varieties (Balesdent et al., 2002, 2013), while *Rlm5* and *RlmJ1* are found in *B. juncea* (genome AABB), though it has not yet been determined which genome they reside in or if these in fact represent the same gene (Balesdent et al., 2002; van de Wouw et al., 2014). Several other mapping efforts have localized resistance loci (*LEM1, LmFr1, LmR1, ClmR1*) to chromosome A07 of *B. napus* (Dion et al., 1995; Ferreira et al., 1995; Mayerhofer et al., 1997, 2005), though it has been assumed that these all correspond to *Rlm4* based on map position and phenotypic response (Rouxel et al., 2003b; Rimmer, 2006).

Our understanding of *Brassica*—*Leptosphaeria* interactions has expanded tremendously over the last decade, aided greatly by the availability of both pathogen (Rouxel et al., 2011) and host (Wang et al., 2011; Chalhoub et al., 2014; Liu et al., 2014) genomes. To date, eight *R* gene-interacting effectors have been cloned from *L. maculans*; *AvrLm1, AvrLm2, AvrLm3, AvrLm4-7* & *AvrLm7, AvrLm6, AvrLm11, AvrLmJ1* (Gout et al., 2006; Fudal et al., 2007; Parlange et al., 2009; Balesdent et al., 2013; van de Wouw et al., 2014; Ghanbarnia et al., 2015; Plissonneau et al., 2016), along with two of the corresponding host *R* genes; *LepR3* and *Rlm2* (Larkan et al., 2013, 2015). Yet despite the rapid expansion of genetic information available for the pathosystem, most characterisations of fungal isolates and host germplasm still rely almost solely on pathological studies for generating *Avr* and/or *R* gene hypotheses. The differential host germplasm used in pathology-based studies often varies between labs, can contain multiple known *R* genes and is sometimes poorly characterized in terms of homozygosity, confirmation of the genetic interactions and the non-specific resistance effects observed during the interaction.

Here we describe the production of single-*R* gene *B. napus* introgression lines (ILs) for use in the pathological and molecular study of *Brassica*—*Leptosphaeria* interactions. Each IL contains one of seven blackleg resistance (*R*) genes (*Rlm1, Rlm2, Rlm3, Rlm4, LepR1, LepR2* & *LepR3*) introgressed into a common susceptible *B. napus* line (Topas DH16516) allowing for the study of *Avr*-*R* gene interactions with a minimum of phenotypic ambiguity.

## Materials and methods

### Plant material

*B. napus* parental lines used in the production of the ILs (Topas, Topas DH16516, Quinta DH24288, Glacier DH24287, Scoop, 1065, 1135, and Surpass 400—Supplementary Table 1) were sourced from collections held by the Rimmer, Lydiate and Borhan labs, AAFC Saskatoon. Doubled-haploid (DH) line Topas DH16516 was originally produced by G. Séguin-Swartz, AAFC Saskatoon. DH lines Quinta DH24288 and Glacier DH24287 were produced by S.R. Rimmer, AAFC Saskatoon, from Quinta and Glacier variety seed originally provided by P.H. Williams, University of Wisconsin. Additional *B. napus* variety lines were sourced from the Lydiate and Vail Labs, AAFC Saskatoon.

### Fungal material

*L. maculans* isolates 98-15s, 00-100s, WA30s, 05-31s, and 2367s were produced from single-spore re-isolations of stock isolates contained in the Rimmer Collection, AAFC Saskatoon. Isolate v23.1.3 (Rouxel et al., 2011) was provided by T. Rouxel, INRA-Bioger, France. Isolate B14-13s was isolated from infected field stubble, provided by F.L. Dokken-Bouchard and D.T. Stephens, Saskatchewan Ministry of Agriculture, Canada, collected during the 2014 Saskatchewan Disease Survey (Dokken-Bouchard et al., 2015). Isolate 3R11 was provided by A. Van der Wouw and B. Howlett, University of Melbourne, Australia. Six of the isolates (98-15s, 00-100s, WA30s, 89-12s, 2367s, and 3R11s) have previously been fully-characterized via whole-genome resequencing (Ghanbarnia et al., 2015). Transgenic isolates 3R11:*AvrLm1* (Larkan et al., 2013), 2367:*AvrLm4-7* and 2367:*AvrLm7* (Larkan et al., 2016) have been described previously, as has the genomic *AvrLm2* construct used to produce 3R11:*AvrLm2* (Ghanbarnia et al., 2015). The remaining transgenic isolates (2367:*AvrLm3* and 3R11:*AvrLm6*) were produced by introducing genomic avirulence allele constructs, including approximately 1 kb 5′ and 500 bp 3′ of the CDS (Supplementary Table 2) using the same *Agrobacterium* transformation method (Utermark and Karlovsky, 2008) and the fungal transformation vector (Larkan et al., 2013) as described previously.

### Production of introgression lines

Initially F_1_ seed was produced by first crossing the resistant donor parent lines (Supplementary Table 1) to either the susceptible Topas variety line (for *Rlm1, Rlm2*, and *Rlm3* populations) or the DH Topas DH16516 (*Rlm4, LepR1, LepR2*, and *LepR3*) once this line became available. F_1_ seedlings were assayed for resistance to *L. maculans* isolates previously determined to be avirulent toward the targeted *R* gene. A single resistant F_1_ selection was then backcrossed to the susceptible Topas variety or Topas DH16516 line (once this line became available) to produce BC_1_F_1_ seed. All backcross generations from BC_2_F_1_ onward were produced using Topas DH16516 exclusively, for all lines. Recurrent backcrossing and selection, both for resistance and spring-type growth habit (where applicable), was performed to produce BC_5_F_1_ seedlings, which were selfed through three additional generations to produce BC_5_F_4_. At this point the lines were tested for homozygous resistance and a final single-plant selection was made. Where the donor parent line contained multiple *R* genes, duel-isolate tests were performed at either BC_1_F_1_ or BC_2_F_1_ to select for individuals that only contained the targeted gene.

### Genetic mapping of A07 *R* gene loci

Genetic mapping of three *R* gene loci (*Rlm1, Rlm3, Rlm4*) was performed in parallel to the production of the ILs by the previously-described methodology (Larkan et al., 2014). Briefly, large BC_1_F_1_ populations (Supplementary Table 1) were produced from the same F1s used for the IL development. Small populations were used to establish linkage of phenotype to simple sequence repeat (SSR) markers positioned on A07 and to assemble draft maps. SSR markers flanking the target genes were then used to screen the full population. Individual BC_1_F_1_ seedlings containing recombination events within the targeted *R* loci regions were reserved for the fine mapping study. The initial genotypes for these ‘recombinants’ were confirmed using DNA from a second round of extractions, and phenotypes were confirmed by examining the segregation of resistance in the BC_1_F_2_ generation. Additional mapping using smaller BC_1_F_1_ populations was later performed to obtain the genomic positions of *Rlm7* and *Rlm9* (donor parental lines Roxet and Goéland, respectively) relative to the other A07 *R* genes (*Rlm1, Rlm3*, and *Rlm4*) and to facilitate the future production of additional ILs.

### Phenotypic verification

To demonstrate the phenotypic reaction of the ILs when challenged with *L. maculans*, seven well-characterized isolates of varying genotypes (*Avr* complements) where chosen from the lab's collection and used to inoculate wounded cotyledons of each line, after which the pathological interaction was rated on a continuous 1 to 9 scale as previously described (Larkan et al., 2013). In parallel to the IL testing the resistant donor parent lines and several other *B. napus* lines previously used to assess *Brassica*—*Leptosphaeria* interactions (Balesdent et al., 2001, 2002, 2005; Rouxel et al., 2003a,b; Stachowiak et al., 2006; Parlange et al., 2009; Yu et al., 2012; Larkan et al., 2013, 2014, 2015) were also tested. Eight seedlings of each line were used in the test, with one inoculation per cotyledon lobe (4 per seedling). Interactions were scored at 10 and 14 days post-inoculation (dpi) with each lesion being rated independently (32 lesions/line). Phenotyping for the disambiguation of *Rlm3* and *Rlm4* in *B. napus* cultivars was performed as described above using 4 seedlings per line (16 inoculations), with phenotypic rating at 14 dpi. Additional confirmation of *R* gene content in each line was produced using transgenic *L. maculans* isolates carrying the characterized *Avr* genes *AvrLm1* (Gout et al., 2006), *AvrLm2* (Ghanbarnia et al., 2015), *AvrLm3* (Plissonneau et al., 2016), *AvrLm4-7* and *AvrLm7* (Parlange et al., 2009), and *AvrLm6* (Fudal et al., 2007).

### Whole-genome SNP analysis

In order to determine the size and genomic location of the donor parent genomic introgressions which harbor each of the targeted *R* genes, as well as any other significant introgressions elsewhere in the genome of each IL, whole-genome SNP analysis was performed using Infinium SNP arrays (Illumina) containing approx. 52, 000 SNPs for the Brassica A and C genomes (Clarke et al., 2016). This also served to determine the level of polymorphism between Topas DH16516 and the donor parent lines and to assess the overall homogeneity of each IL. To perform the analysis, DNA was extracted as previously described (Larkan et al., 2013) from 218 individual *B. napus* plants; 20 replicates of each IL line, six replicates of Topas DH16516, three replicates of original Topas variety line and single replicates of each donor parent and several other common blackleg differential lines were also included in the analyses (Supplementary Table 3). Sample were analyzed in two separate Infinium runs. To minimize spurious genotyping, strict criteria were imposed for the selection of high-quality SNP markers. All SNPs were initially clustered using Genome Studio software (Illumina) and markers with 100% heterozygous calls (33) were removed. Further quality control was performed by the removal of markers determined to be skewed, potentially detecting SNPs at more than one locus, based on (a) the polar co-ordinates of genotypic clusters (AA T Mean > 0.35; BB T Mean <0.65; AB T Mean <0.2, >0.8) and (b) cluster separation (<0.2). All markers with a call frequency > 0.091 (at least two samples called) were exported to Microsoft Excel for further sorting. Approximately 600 additional markers were removed due to inconsistencies between Infinium runs [usually low signal markers called as heterozygous (AB) for one run, null for the other]. In total 43,211 SNP markers (82.8%) were retained for use in assessing polymorphism between *B. napus* lines and to define the donor parent introgressions in each IL.

## Results

### Genetic mapping

**Figure 1:**
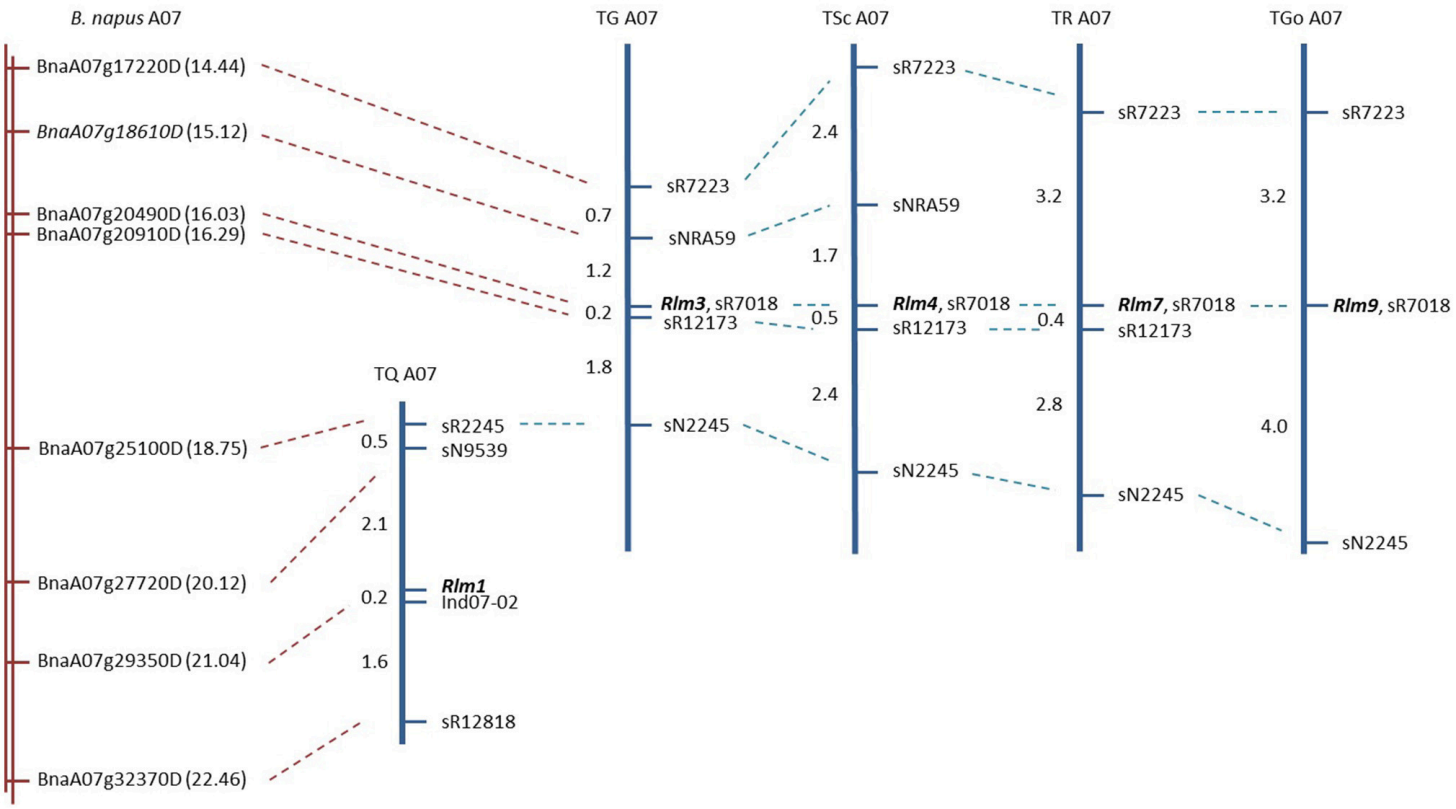
**Physical and genetic maps of five A07 blackleg *R* genes**. Portion of reference *B. napus* Darmor-*bzh* genome (Chalhoub et al., 2014) corresponding to mapped locations of *R* genes shown in red. Gene names (BnaA07g…) represent closest gene to corresponding marker, indicated by dashed red line. Gene positions (Mb) given in brackets. Dashed blue lines indicate common markers, mapped *R* gene names in bold. Genetic distance between markers given in centiMorgans.

Genetic mapping of five *R* gene loci (*Rlm1, Rlm3, Rlm4, Rlm7*, and *Rlm9*) was performed to confirm the genes being introgressed (in the case of *Rlm1, Rlm3*, and *Rlm4*) were consistent with the originally-described chromosome A07 *R* loci (Delourme et al., 2004), to determine the physical positions of the genes in the *B. napus* genome (Chalhoub et al., 2014), and to complement our own previously-described fine mapping of *Rlm2* (Larkan et al., 2014), *LepR1* and *LepR2* (Yu et al., 2012) and *LepR3* (Larkan et al., 2013). All five genes were determined to reside within the mapped region of chromosome A07, which was defined by SSR markers spanning approximately 8 Mb of the *B. napus* “Darmor-*bzh*” genome (Figure 1). Four of the *R* genes (*Rlm3, Rlm4, Rlm7*, and *Rlm9*) were found to be tightly clustered as previously described (Delourme et al., 2004); all four genes co-segregated with the SSR marker sR7018, which corresponds to chrA07:16027226.16027883 of the *B. napus* Darmor-*bzh* genome (nearest gene BnaA07g20490D). *Rlm3* and *Rlm4* were both localized within an interval of approximately 1.2 Mb containing 230 predicted genes. The mapping of *Rlm7* and *Rlm9* placed these *R* loci within intervals of 1.85 Mb (369 genes) and 4.31 Mb (788 genes), respectively. The remaining A07 *R* gene, *Rlm1*, was localized to a region 3.8 Mb below the *Rlm3*/*Rlm4* map interval, positioned in an interval of approximately 920 kb (163 predicted genes) between the markers sN9539 and Ind07-02 (Figure 1). The physical location for *Rlm3* and *Rlm4* are in agreement with our own previous lower-resolution mapping of these genes (Larkan et al., 2016).

### Whole-genome SNP analysis

After sorting of the 43,211 SNP markers to distinguish homozygous (AA or BB), heterozygous (AB) and null (no call) SNP alleles for both Topas DH16516 and *R* gene donor parent lines, over-all polymorphism relative to Topas DH16516 was analyzed, calculated as a mean of each IL replication, with ILs ranging from 92.9 to 98.9% identical to Topas DH16516 in terms of SNP polymorphism (Supplementary Table 3). Surprisingly, the Topas variety line originally used to establish backcross populations for Topas-*Rlm1*, Topas-*Rlm2* and Topas-*Rlm3* was found to share only 82.7% marker identity with Topas DH16516. To further define the genomic arrangements of the ILs, SNPs were ordered based on chromosome position (based on Darmor reference chromosome builds spanning 643,396,264 bp over 19 chromosomes) and surveyed for regions of significant variation from Topas DH16516 (>3 clustered markers) to define the donor parent introgressions and null regions carried in each line. Each IL was found to carry a donor parent chromosomal introgression corresponding to the mapped *R* locus for each gene, ranging in size from 925 kb to 13.95 Mb, with most *R* gene introgressions spanning less than 5 Mb (Supplementary Table 4). Several other donor parent introgressions were present as both fixed and segregating genomic segments, including several remnants of the original Topas variety line in the first three ILs. A summary of the total significant donor parent introgressions along with significant null regions is presented in Table 1. The genomic position of each introgression relative to the Darmor-*bzh* reference genome is presented in Supplementary Table 4. Production of the three ILs carrying A07 *R* gene introgressions (Topas-*Rlm1*, Topas-*Rlm3*, Topas-*Rlm4*) appeared to be affected by a common A07-C06 reciprocal translocation event as a result of ancestral homology between the Brassica A and C genomes (Osborn et al., 2003). This event is likely carried by the Topas DH16516 parental line, which has resulted in null regions (12.6 Mb) for the affected ILs on lower C06 and corresponding dense marker heterozygosity on the homoeologous region of lower A07.

**Table 1:** **Significant non-Topas DH16516 variation in each Introgression Line**.

	**Fixed**	**Segregating**	**Null**	**Total**	**Non-DHT (%)**
Topas-*Rlm1*	14,089,509	389,468	17,080,797	31,559,774	4.91
Topas-*Rlm2*	7,099,003	6,060,940	0	13,159,943	2.05
Topas-*Rlm3*	68,137,781	4,906,588	12,607,348	85,651,717	13.31
Topas-*Rlm4*	14,771,707	30,755,969	12,607,348	58,135,024	9.04
Topas-*LepR1*	10,428,616	21,356,340	0	31,784,956	4.94
Topas-*LepR2*	15,787,088	1,138,183	0	16,925,271	2.63
Topas-*LepR3*	9,433,113	1,263,529	0	10,696,642	1.66

Total estimated donor parent genomic content (bp) present in each IL as fixed, segregating or null genomic introgressions based on whole-genome SNP analysis.

### Pathology

**Figure 2:**
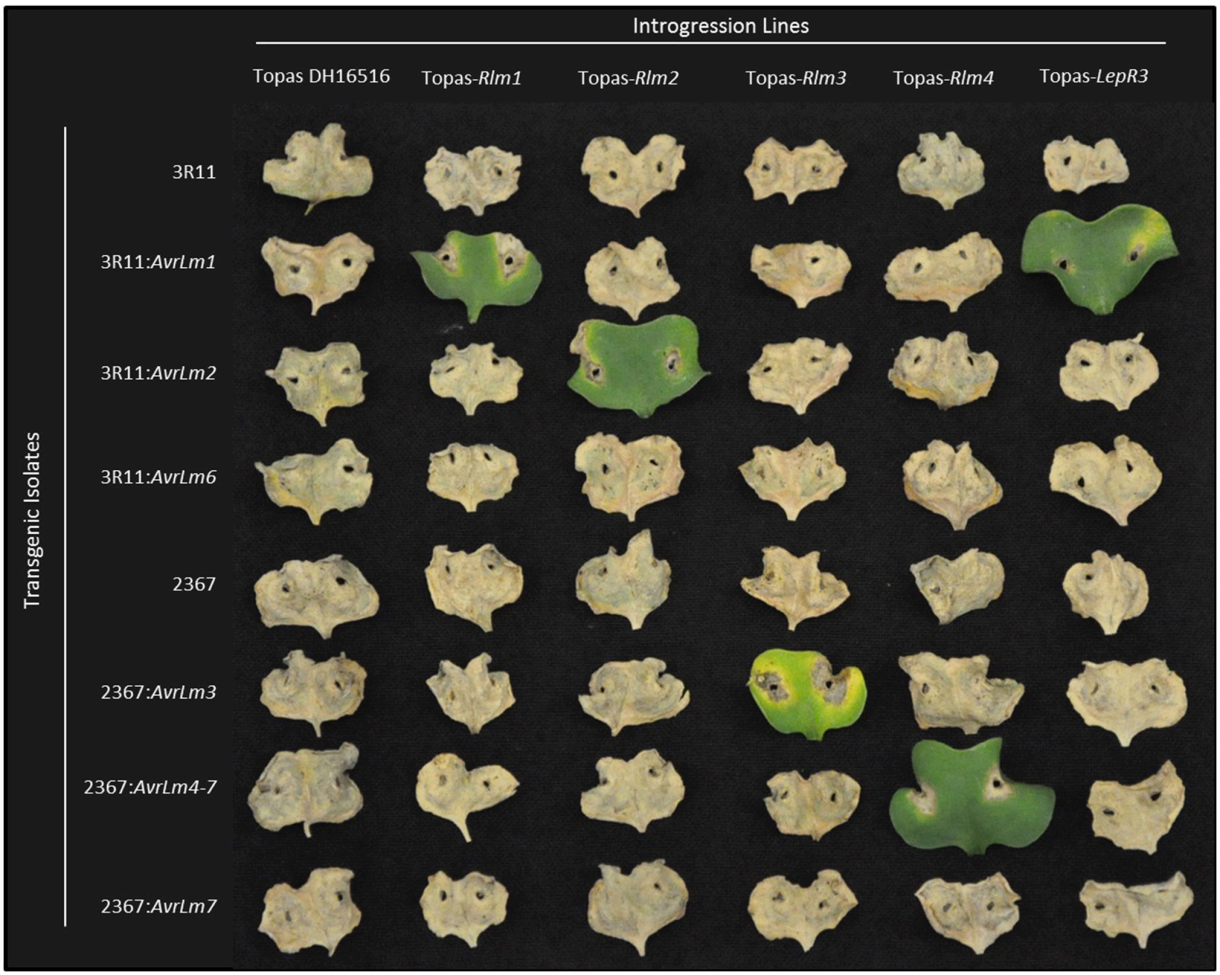
**Confirmation of introgression line *R* gene content though transgenic complementation of avirulence determinants**. Cotyledon reactions of susceptible *B. napus* line Topas DH16516 and five ILs challenged with two native *L. maculans* isolates (3R11 & 2367) and six transgenic isolates carrying additional *Avr* gene constructs. Cotyledons shown at 14 days post-infection.

Average ratings for each *B. napus* line at each time point (10 and 14 dpi) on a scale of 1 to 9 (with 9 being fully susceptible) are presented in Table 2. For the ILs, clear differentials were observed between avirulent (*Avr*) and virulent (*avr*) isolates at 10 dpi, with isolates reaching full infection (mean rating score >8) on the susceptible lines by 14 dpi. For incompatible interactions, mean ratings ranged between 2.1 to 4.1 for all ILs except Topas-*LepR2*, for with scores ranged 3.1 to 4.8. Interactions for *Rlm1, Rlm2, Rlm3, Rlm4*, and *LepR3* were all confirmed using transgenic isolates carrying the corresponding *Avr* gene (Figure 2). Results were consistent with previous pathological testing conducted with these and other *L. maculans* isolates over several years.

**Table 2:** **Phenotypic characterisation of *L. maculans* resistance response for ILs, donor parents and other *B. napus* controls**.

***L. maculans* Isolates^a^**	***B. napus* Lines^b^**																										
	**dpi**	**Topas DH16516**	**Topas-*Rlm1***	**Topas-*Rlm2***	**Topas-*Rlm3***	**Topas-*Rlm4***	**Topas-*LepR1***	**Topas-*LepR2***	**Topas-*LepR3***	**Westar**	**Quinta DH24288**	**Columbus**	**Glacier DH24287**	**Bristol**	**NLA51-1**	**Quantum**	**AG-Castle**	**Scoop**	**Jet Neuf**	**Falcon**	**Roxet**	**Goéland**	**Darmor**	**1065**	**1135**	**Surpass 400**	**NLA8-2**
		–	*Rlm1*	*Rlm2*	*Rlm3*	*Rlm4*	*LepR1*	*LepR2*	*LepR3*	*-*	*Rlm1*	*Rlm1*	*Rlm2*	*Rlm2*	*Rlm2*	*Rlm3*	*Rlm3*	*Rlm4*	*Rlm4*	*Rlm4*	*Rlm7*	*Rlm9*	*Rlm9*	*LepR1*	*LepR2*	*LepR3*	*LepR3*
											*Rlm3*	*Rlm3*	*Rlm3*	*Rlm9*												*RlmS*	
**v23.1.3**	10	7.9	2.7	7.9	7.1	2.9	2.5	6.5	1.4	8.0	3.6	2.5	7.2	7.7	7.2	6.7	7.9	1.2	2.6	2.9	3.5	7.0	7.5	2.8	7.9	2.5	1.8
A1-A4-(5)-A6-A7-(8)-(10)-11*-J1*-(S)-AL1-AL3	14	9.0	3.0	8.9	9.0	2.9	3.5	8.2	2.1	9.0	5.2	3.6	8.9	8.9	9.0	8.8	8.8	1.8	2.9	3.2	4.4	9.0	9.0	5.9	5.6	3.6	2.2
**98-15s**	10	7.8	8.0	3.0	8.0	2.9	2.8	3.1	7.6	7.8	8.0	7.4	3.1	3.9	2.3	6.5	7.0	2.6	2.8	2.7	3.3	7.9	7.6	3.3	6.6	4.3	8.2
A2-A4-(5)-A6-A7-(8)-(10)-11*-J1*-(S)-AL1-AL2	14	9.0	9.0	3.2	9.0	3.0	3.7	3.8	9.0	9.0	9.0	9.0	3.7	4.8	3.0	8.7	8.3	2.7	2.9	2.8	3.3	8.7	8.5	7.0	8.1	6.9	9.0
**00-100s**	10	7.9	8.3	2.3	2.5	7.9	2.4	3.0	8.0	7.9	3.0	2.5	2.2	2.8	1.7	2.4	1.8	7.8	6.3	6.3	6.7	3.3	3.3	2.9	4.2	2.8	8.0
A2-A3-(5)-A6-(8)-A9-(10)-J1*-AS-AL1-AL2	14	8.8	9.0	2.8	3.2	8.9	2.9	3.1	8.9	9.0	4.0	3.4	2.5	3.0	2.6	3.0	2.7	9.0	8.0	7.4	7.8	3.9	4.1	3.2	5.5	3.1	9.0
**WA30s**	10	7.8	7.8	7.0	3.0	7.3	3.1	3.5	7.0	7.3	3.3	2.7	3.1	6.5	7.1	2.5	2.2	7.4	4.8	6.4	6.8	6.5	6.8	3.0	4.7	3.2	7.1
A3-(5)-A6-(8)-(10)-J1*-AS-AL1-AL2	14	8.8	9.0	5.6	3.3	8.9	3.3	4.0	8.9	8.4	3.8	3.0	3.7	9.0	8.8	2.9	3.0	9.0	7.6	8.8	8.2	5.6	7.1	3.5	5.4	3.2	9.0
**89-12s**	10	7.0	7.2	7.1	7.0	7.2	2.9	4.1	7.0	7.3	7.2	7.1	6.0	4.7	7.0	6.8	7.2	7.0	5.4	6.3	6.9	3.3	4.2	2.4	6.2	3.1	7.0
(5)-A6-(8)-A9-(10)-11*-J1*-AS-AL1-AL2	14	9.0	9.0	9.0	9.0	8.9	3.2	4.5	9.0	8.9	8.8	9.0	8.3	5.7	8.9	8.8	5.6	9.0	7.9	8.3	7.9	3.9	5.0	4.1	7.3	3.1	9.0
**05-31s**	10	7.6	8.1	3.0	7.8	7.1	3.3	4.0	7.0	8.0	8.0	8.0	3.1	3.3	2.9	7.6	7.3	7.1	4.1	4.7	3.5	6.8	7.6	6.3	6.3	3.4	7.6
A2-(5)-A6-A7-(8)-(10)-11*-J1*-AS-AL1-AL2	14	9.0	9.0	3.4	9.0	8.3	4.1	4.6	9.0	9.0	8.9	9.0	4.1	3.8	3.1	8.9	9.0	9.0	7.1	7.3	4.0	8.2	8.8	8.4	7.7	3.9	9.0
**B14-13s**	10	7.7	8.1	3.0	7.8	7.6	8.0	3.9	8.2	8.1	8.2	8.1	3.0	3.0	2.9	7.2	7.3	8.0	4.8	4.9	5.9	3.3	4.3	8.0	6.7	3.1	8.0
A2-(5)-A6-(8)-A9-(10)-11*-J1*-AS-AL2	14	9.0	9.0	3.2	9.0	9.0	9.0	4.4	9.0	9.0	9.0	8.9	3.7	3.0	3.0	8.9	8.4	9.0	6.4	6.4	7.5	3.9	5.1	9.0	7.7	3.1	9.0
**3R11s**	10	6.7	7.0	6.8	6.9	6.8	3.1	4.2	6.9	7.3	7.0	6.8	6.5	7.0	6.9	7.3	7.1	7.0	5.6	5.5	4.0	6.9	6.9	4.1	5.9	4.2	7.0
A7-11*-J1*-AS-AL1-AL2	14	9.0	9.0	9.0	9.0	8.9	3.4	4.8	9.0	8.8	8.8	9.0	8.9	9.0	9.0	9.0	9.0	9.0	8.0	8.3	4.5	8.5	8.3	7.0	9.0	6.6	9.0
**3R11:*****AvrLm1***	10	6.5	3.0	7.0	6.9	6.6	3.2	4.1	2.6	7.9	5.3	3.0	6.7	7.0	6.9	7.1	7.0	7.0	5.3	6.2	4.6	6.9	7.1	5.1	5.8	3.5	2.0
A1-A7-11*-J1*-AS-AL1-AL2-AL3	14	8.5	3.2	9.0	8.8	8.3	3.4	4.8	3.0	9.0	7.0	4.6	5.6	8.9	9.0	9.0	9.0	9.0	7.9	9.0	5.3	9.0	8.8	7.5	9.0	4.2	2.7
**3R11:*****AvrLm2***	10	6.8	6.9	2.8	6.8	6.6	3.0	3.8	6.1	7.0	6.9	6.8	2.8	3.0	2.4	6.9	7.1	7.0	5.3	6.6	3.8	6.4	6.7	3.5	4.9	4.2	7.0
A2-A7-11*-J1*-AS-AL1-AL2	14	9.0	9.0	2.9	9.0	8.3	3.6	4.4	9.0	8.8	8.5	9.0	3.0	3.1	2.9	9.0	9.0	9.0	7.8	7.6	4.1	8.1	8.1	6.4	8.8	5.8	9.0
**3R11:*****AvrLm6***	10	6.9	7.0	6.9	7.0	6.1	3.0	3.3	6.6	6.9	7.0	7.0	7.0	7.0	7.0	6.9	7.0	7.0	5.4	6.4	3.5	6.4	6.9	3.3	4.5	4.3	7.0
A6-A7-11*-J1*-AS-AL1-AL2	14	9.0	9.0	9.0	9.0	5.6	3.4	3.9	9.0	8.5	8.8	9.0	8.5	9.0	9.0	9.0	9.0	9.0	7.1	8.0	3.8	8.0	8.8	5.8	8.8	6.9	9.0
**2367s**	10	7.3	7.3	7.0	7.0	6.9	3.0	3.4	6.7	6.6	7.4	6.5	6.3	7.3	7.0	6.9	7.0	7.0	5.4	6.6	7.1	6.9	6.9	3.4	5.6	3.3	7.0
A6-11*-J1*-AS-AL1-AL2	14	8.9	9.0	9.0	9.0	8.9	3.3	4.3	5.6	8.1	9.0	8.8	8.0	9.0	9.0	9.0	9.0	9.0	8.0	9.0	8.7	8.8	5.6	5.3	7.7	4.8	9.0
**2367:*****AvrLm3***	10	7.1	7.3	6.9	2.9	7.2	2.9	3.4	6.8	6.7	3.2	2.8	3.1	7.0	6.8	3.3	2.9	7.0	5.2	6.8	7.1	5.9	6.9	3.0	5.3	3.1	7.0
A3-A6-11*-J1*-AS-AL1-AL2	14	9.0	9.0	8.9	3.9	9.0	3.4	4.4	8.7	8.5	4.1	3.0	4.3	9.0	9.0	3.3	3.1	9.0	7.5	9.0	8.8	8.5	8.7	3.7	8.4	4.6	9.0
**2367:*****AvrLm4-7***	10	7.3	7.0	7.0	7.0	3.0	3.1	3.8	7.3	7.1	7.5	6.4	6.7	7.2	7.0	7.1	7.0	3.0	2.8	3.0	3.1	7.0	6.9	3.0	6.8	4.8	7.1
A4-A6-A7-11*-J1*-AS-AL1-AL2	14	9.0	9.0	8.9	9.0	3.1	3.6	4.6	9.0	5.6	9.0	8.5	8.8	8.9	9.0	9.0	9.0	3.3	2.9	3.0	3.7	8.8	8.8	4.4	8.7	5.9	9.0
**2367:*****AvrLm7***	10	6.9	7.1	7.0	7.0	6.4	3.0	3.5	7.1	6.8	7.5	6.8	6.6	7.0	6.9	6.9	7.0	7.0	5.8	6.6	3.0	7.0	7.1	3.1	6.3	5.0	7.0
A6-A7-11*-J1*-AS-AL1-AL2	14	9.0	9.0	8.9	9.0	8.5	3.6	4.6	9.0	5.6	9.0	8.5	8.8	9.0	9.0	9.0	9.0	9.0	7.3	8.9	3.2	9.0	8.8	4.8	8.0	6.6	9.0

Shaded numbers represent averaged phenotypic rating (0–9 scale), dark green, most resistant; dark red, most susceptible,

a native and transgenic L. maculans isolates followed by pathotype (A1–AS, AL1–AL3 = avirulent against Rlm1–RlmS, LepR1–LepR3, numbers in brackets denote interactions not determined, asterix denotes predicted phenotype based on genotyping, underlined entries denote avirulence conveyed by transgenic construct),

b B. napus lines followed by known blackleg R gene content, bold entries denote introgressed R gene source.

**Figure 3:**
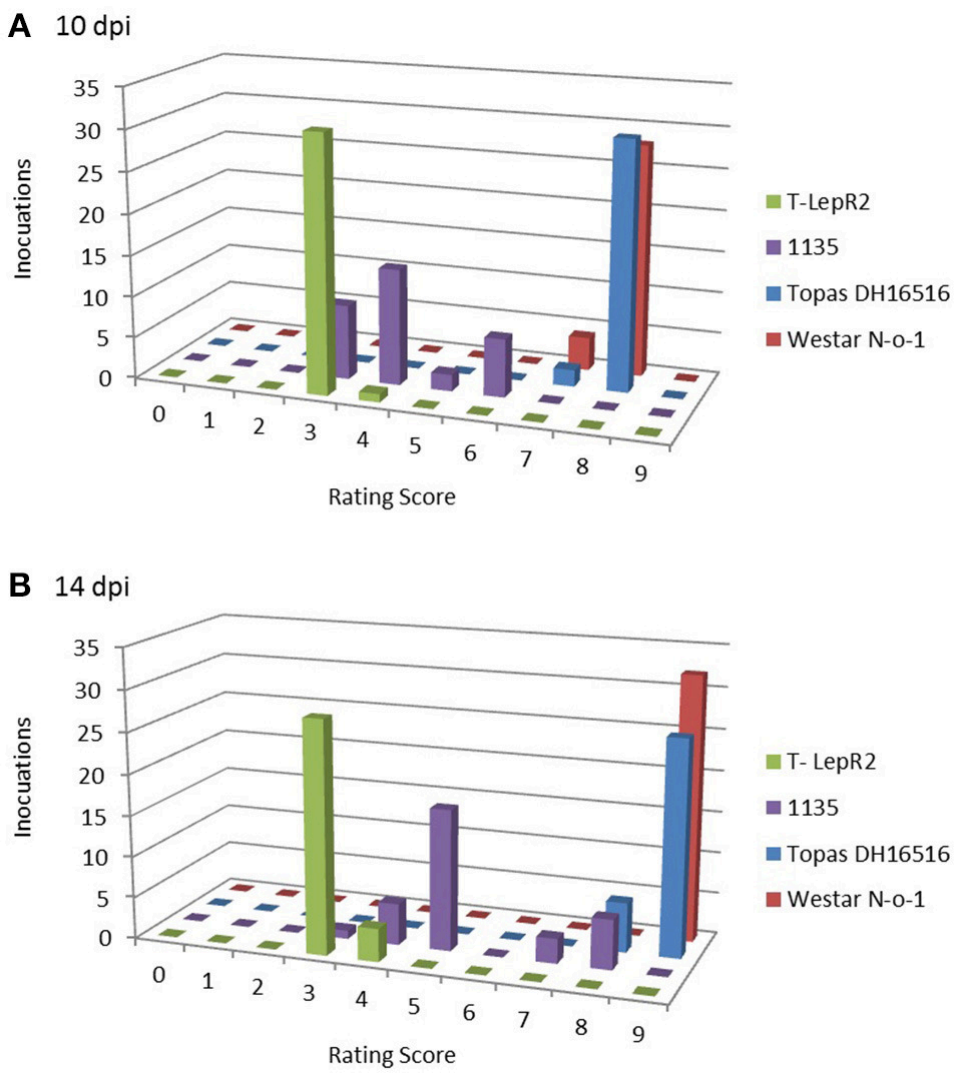
**Distribution of cotyledon lesion ratings for *LepR2* lines Topas-*LepR2* and 1135**. Vertical bars represent number of lesions scored for each rating scale graduation (0, no infection; 9, complete tissue collapse), total 32 lesions per line, at **(A)** 10 days post-inoculation (dpi) and **(B)** 14 dpi, when challenged with *L. maculans* isolate 00–100.

The clear differential between susceptible and resistant reactions observed for the ILs was not true for several of the other control lines tested, with incompatible interactions resulting in intermediate to mildly susceptible mean rating scores. For example, the *Rlm1* line Quinta DH24288 (donor parent of the Topas-*Rlm1* IL) produced an intermediate score of 5.2 when challenged with the *AvrLm1* isolate v23.1.3, and ratings progressed from 5.3 to 7.0 between 10 and 14 dpi when inoculated with the transgenic isolate 3R11:*AvrLm1*. This weak response was not mirrored by another *Rlm1* variety, Columbus, which resulted in mean scores of 3.6 and 4.6 when challenged with v23.1.3 and 3R11:*AvrLm1*, respectively. While all isolates, with the exception of B14-13s, were clearly avirulent on the IL Topas-*LepR1*, several isolates produced intermediate or susceptible scores (5.3 to 8.4) on the *LepR1* donor line 1065. The most dramatic difference between IL and donor line and was observed for Topas-*LepR2* and 1135. While all isolates except v23.1.3 were avirulent on Topas-*LepR2* at 14 dpi (3.1 to 4.8), all isolates were rated as intermediate to susceptible (5.4 to 9) on the *LepR2* donor parent 1135. Though clear resistance reactions (scores <5) were observed for at least a few individual inoculation points on 1135 cotyledons for most isolates, a wide distribution of rating scores was observed, usually resulting in an overall susceptible rating. Even isolates that produced intermediate averaged rating scores, such as 00-100s, a broad range of individual lesion ratings, including several highly-infected lesions (score 7–8), were observed. This volatility was not observed for Topas-*LepR2* which displayed a much more consistent resistance response (Figure 3).

In phenotypic comparisons between ILs and the other *B. napus* control lines, restriction of lesions in some *B. napus* lines was observed which were not associated with known *Avr*-*R* gene interactions, particularly for the variety line Jet Neuf carrying *Rlm4*. All *Rlm4*-virulent (*avrLm4*) isolates were slower to establish infection on Jet Neuf than on other susceptible lines (10 dpi scores 4.1–6.3) and some weak HR was observed around the infection sites. These lesions failed to reach full infection (<8) by 14 dpi. Similar, though less severe restriction of infection was also observed for the varieties Falcon and Roxet (Supplementary Figure 1).

In additional testing, 31 *B. napus* variety lines held within AAFC collections, many of which had previously been assessed for use in differential *L. maculans* pathology testing and designated as *Rlm3* or *Rlm4* varieties, were assayed for the presence of *Rlm3* and *Rlm4* using the same isolates used to define the IL interactions (v23.1.3, 98-15s, 00-100s, WA30s, 89-12s, 05-31s, B14-13s, and 2367s), along with the transgenic isolates 2367:*AvrLm3*, 2367:*AvrLm4-7* and 2367:*AvrLm7*. Topas DH16516, Topas-*Rlm3* and Topas-*Rlm4* were included as controls. Eighteen lines were confirmed as carrying *Rlm3* by their interactions with both native *AvrLm3* isolates (00-100s and WA30s) and the transgenic isolate 2367:*AvrLm3* (Table 3). These 18 lines included six lines previously designated as *Rlm4* varieties (Major, Crésor, Shiralee, Maluka, DH12075 and Range) and 10 lines which had not previously been characterized or had been deemed as “susceptible” varieties. Seven lines were confirmed to carry *Rlm4*, including two varieties not previously defined as *Rlm4* varieties. Of particular note is the Australian variety Trigold, which is included as a susceptible control in some field-based disease nurseries (Raman et al., 2012b; Larkan et al., 2016). No lines were detected to carry both *Rlm3* and *Rlm4*, though Tapidor DH, which we have previously shown to carry *Rlm2* (Larkan et al., 2015), appears to additionally contain *Rlm9*. Similar to observations made during the characterisation of the ILs, *B. napus* variety lines carrying the same *R* gene (in this case *Rlm3*) displayed wide variance in the amplitude of HR after infection with *AvrLm3* isolates. For example, variety Range produced highly resistant averaged ratings (1.6 to 2.9) while the average scores for AC-Excel were graded as intermediate to mildly susceptible (4.6 to 6.0). Rating scores for the *Rlm4* varieties appeared to be more consistent (2.4 to 3.8) though several lines, including Dunkeld and AV-Sapphire, showed similar non-*Avr* associated restriction of lesions as previously observed for Jet Neuf. This was also apparent in the *Rlm2, Rlm9* lines Samourai and Tapidor DH (Table 3).

**Table 3:** **Determination of *Rlm3* and *Rlm4* in *B. napus* varieties**.

	***L. maculans* Isolate^b^**													
			**00-100s**	**v23.1.3**	**98-15s**	**WA30s**	**89-12s**	**05-31s**	**B14-13s**	**2367s**	**2367:*AvrLm3***	**2367:*AvrLm4-7***	**2367:*AvrLm7***	
***B. napus* Line^a^**	**Reported *R* gene**	**Reference**												**Determined *R* gene^c^**
Topas DH16516	none	1	8.8	9.0	9.0	8.8	9.0	9.0	8.9	8.9	9.0	9.0	9.0	none
Topas-*Rlm3*	*Rlm3*	this study	3.2	9.0	9.0	3.3	9.0	9.0	9.0	9.0	3.9	9.0	9.0	*Rlm3*
Topas-*Rlm4*	*Rlm4*	this study	8.9	2.9	3.0	8.9	8.9	8.3	9.0	8.9	9.0	3.1	8.5	*Rlm4*
Range	*Rlm4*	2	2.0	8.9	8.6	1.6	9.0	9.0	8.8	8.8	2.9	8.8	8.8	*Rlm3*
Maluka	LmR1 (*Rlm4*)	2,3,4	2.1	8.9	8.1	3.2	9.0	9.0	8.9	8.5	3.0	9.0	8.8	*Rlm3*
Yickadee	none	2	2.1	9.0	8.1	2.0	8.8	9.0	9.0	8.5	3.0	8.2	8.8	*Rlm3*
Q2	*Rlm3/Rlm3 + Rlm9*	5,6,7,8	2.8	8.8	8.9	3.1	8.8	9.0	9.0	8.5	3.6	9.0	9.0	*Rlm3*
Shiralee	LmR1 (*Rlm4*)	3,4,9	3.0	9.0	9.0	2.5	9.0	9.0	9.0	9.0	3.4	9.0	9.0	*Rlm3*
Dac-1	unknown	10,11	3.0	8.4	8.8	2.2	9.0	9.0	9.0	9.0	3.3	9.0	9.0	*Rlm3*
46A65	n/a	-	3.2	8.8	9.0	3.0	9.0	9.0	9.0	9.0	4.1	9.0	9.0	*Rlm3*
SP-Banner	n/a	-	3.4	9.0	9.0	2.9	8.9	9.0	8.9	9.0	4.2	9.0	9.0	*Rlm3*
Cresor	LmFr1 (*Rlm4*)	4,9,12	3.5	9.0	8.9	2.9	9.0	9.0	9.0	8.8	3.6	8.8	9.0	*Rlm3*
Major	LEM1 (*Rlm4*)	4,13,14	3.7	9.0	9.0	4.0	9.0	9.0	9.0	9.0	4.4	9.0	9.0	*Rlm3*
Defender	n/a	-	4.1	9.0	9.0	3.5	8.9	9.0	9.0	9.0	4.5	9.0	9.0	*Rlm3*
Sprint	n/a	-	4.2	9.0	9.0	3.5	8.4	9.0	9.0	9.0	4.4	9.0	9.0	*Rlm3*
Garrison	n/a	-	4.2	8.8	8.6	3.5	8.6	9.0	9.0	9.0	4.3	9.0	9.0	*Rlm3*
AG-Spectrum	polygenic	15	4.3	9.0	9.0	3.4	9.0	9.0	9.0	9.0	3.9	9.0	9.0	*Rlm3*
DH12075	CLmR1 (*Rlm4*)	9,16	4.4	9.0	9.0	3.8	9.0	9.0	9.0	9.0	4.8	9.0	9.0	*Rlm3*
Sentry	unknown	8	4.6	8.8	9.0	4.5	8.5	9.0	9.0	9.0	4.9	9.0	9.0	*Rlm3*
AC-Excel	n/a	-	6.0	9.0	9.0	5.5	9.0	9.0	9.0	9.0	4.6	9.0	9.0	*Rlm3*
Capitol	*Rlm1, Rlm3*	2,17	3.6	4.9	8.1	2.6	8.4	9.0	8.5	8.8	3.8	9.0	9.0	(*Rlm1/LepR3*), *Rlm3*
Dunkeld	*Rlm4/*poly*/Rpg3Dun*	2,5,15,18,19	8.9	3.0	3.0	7.8	7.8	8.5	9.0	8.5	8.0	2.9	8.0	*Rlm4*
Monty	*Rlm4*	2	8.6	3.0	2.4	8.5	8.8	8.8	8.1	9.0	9.0	3.0	7.8	*Rlm4*
Pollen	*Rlm4*	2,20	8.8	3.0	2.9	8.0	9.0	7.8	8.4	9.0	9.0	3.0	8.8	*Rlm4*
Cooper	*Rlm1, Rlm4*	21	8.6	3.1	3.1	8.2	9.0	8.8	8.8	9.0	9.0	3.0	8.8	(*Rlm1/LepR3*), *Rlm4*
AV-Sapphire	*Rlm4/Rlm4 + Rlm9*	5,15,22	9.0	3.4	3.0	6.8	6.5	8.8	6.9	8.5	8.0	3.1	7.5	*Rlm4*
Trigold	n/a	-	8.5	3.5	3.2	8.5	9.0	9.0	8.5	8.8	9.0	2.6	8.8	*Rlm4*
Val-1	unknown	10,11	9.0	3.8	3.1	8.8	9.0	9.0	9.0	9.0	9.0	3.5	9.0	*Rlm4*
PSA12	n/a	-	9.0	9.0	9.0	8.6	9.0	9.0	9.0	9.0	9.0	9.0	9.0	-
Cadillac	*Rlm1*	23	8.0	4.0	9.0	8.5	8.0	9.0	8.8	9.0	9.0	9.0	9.0	(*Rlm1/LepR3*)
AV-Garnet	*Rlm1, Rlm9*	5	4.8	4.9	9.0	8.4	5.8	9.0	7.7	9.0	9.0	9.0	9.0	(*Rlm1/LepR3, Rlm9*)
Samourai	*Rlm2, Rlm9*	20	2.6	8.2	3.2	7.4	4.2	3.2	2.9	8.2	7.8	8.0	8.4	Rlm2*, (*Rlm9*)
Tapidor DH	*Rlm2/Rlm2 + Rlm4*	2,24	2.0	8.0	2.8	7.9	3.3	3.6	2.9	8.8	8.6	8.8	8.2	Rlm2*, (*Rlm9*)
Avisio	*Rlm9*	25	4.0	9.0	9.0	9.0	4.9	8.8	5.0	8.8	8.5	9.0	8.8	(*Rlm9*)

*Shaded numbers represent averaged phenotypic rating (0–9 scale), dark green, most resistant; dark red, most susceptible*.

a B. napus variety lines and previously-reported R gene content. References: 1, (Larkan et al., 2013); 2, (Rouxel et al., 2003b); 3, (Mayerhofer et al., 1997); 4, (Delourme et al., 2006); 5, (Marcroft et al., 2012); 6, (Van De Wouw et al., 2009); 7, (van de Wouw et al., 2014); 8, (Zhang et al., 2015); 9, (Mayerhofer et al., 2005); 10, (Kutcher et al., 2007); 11, (Moreno-Rico et al., 2001); 12, (Dion et al., 1995); 13, (Ferreira et al., 1995); 14, (Rouxel et al., 2003b); 15, (Raman et al., 2012b); 16, (Yu et al., 2012); 17, (Balesdent et al., 2002); 18, (Li et al., 2004); 19, (Dusabenyagasani and Fernando, 2008); 20, (Balesdent et al., 2005); 21, (Kutcher et al., 2010); 22, (Larkan et al., 2016); 23, (Gout et al., 2006); 24, (Ghanbarnia et al., 2015); 25, (Light et al., 2011).

b Native and transgenic L. maculans isolates, pathotypes as described in Table 2.

c R gene content of each line as determined in this study; genes in brackets not confirmed in this test, asterix denotes genes previously confirmed through cloning (Larkan et al., 2015).

## Discussion

We present here the development and characterisation of single-blackleg *R* gene introgression lines for the study of the *Brassica*–*Leptosphaeria* pathosystem, allowing for the accurate assessment of *Avr*-*R* gene interactions in a highly homogenous genomic background free from ambiguous, phenotype-altering effects present in many other *B. napus* lines. Assessing the *R* gene content of *B. napus* material with varied genomic backgrounds poses several challenges. We noted several varieties which produced mild and inconsistent HR in a non-*Avr* dependent fashion, restricting the spread of infection in some tests. This phenomenon has been noted several times in the past (Rouxel et al., 2003b; Balesdent et al., 2005; Dilmaghani et al., 2009). Specifically, Dilmaghani et al. (2009) noted atypical reactions for tests performed using Jet Neuf which could not be explained by the presence of *AvrLm4* in the affected isolates. Restriction of cotyledon lesions, resulting in less than full susceptibility, has also been observed and associated with adult-plant resistance and field-derived quantitative trait loci (QTL) in several studies (Delourme et al., 2004; Raman et al., 2012b; Huang et al., 2014; Larkan et al., 2016). While inoculation of variety lines affected by this minor restriction of infection still produced 14 dpi rating scores that were regarded as susceptible interactions (>6 on the 0–9 scale), using these lines as differentials under non-ideal conditions, or when using less vigorous inoculum, could lead to an incorrect assessment of either the virulence of the isolate being tested or the *R* gene content of the host line. Phenotyping of *Brassica*—*Leptosphaeria* interactions can also be affected by growth conditions, including temperature and moisture (Huang et al., 2006) and these appear to affect different *R* gene-mediated resistance responses unequally. It should be noted that we did not subject our seedlings to additional humidity to establish infection during the pathological assays, which is a common practice for *L. maculans* pathology. In our experience, *L. maculans* inoculum that fails to reach full infection on Topas DH16516 will also perform poorly on Westar and other susceptible varieties. Inoculum that is sub-optimal may then be subject to further inhibition by non-specific cotyledon resistance found in many lines, leading to a false classification of specific avirulence. The judicious use of susceptible controls is vital to evaluating the interaction of isolates with resistant material. Our results reflect as close to “ideal” conditions for pathotyping as we can achieve.

We also demonstrate here that a range of phenotypic responses can be observed for *B. napus* varieties carrying the same *R* gene. The most striking example of this phenomenon can be seen in the comparison between the IL Topas-*LepR2* and 1135, the *LepR2* donor line (Table 2). 1135 was produced from an initial *B. napus* x *B. rapa* ssp. “sylvestris” (BRS) F_1_, were the *B. napus* parent was DH12075, a Crésor × Westar F_1_-derived DH line (Mayerhofer et al., 2005). 1135 was produced from the DH12075 × BRS F_1_ following successive backcrossing to the DH line Westar N-o-1 before selection at BC_4_ (Yu et al., 2012), so the *LepR2* gene is present in a largely Westar-derived genomic background in this line. In our testing, six of the eight non-transgenic isolates used in the characterisation of the ILs were able to produce mild to severe infection on 1135 cotyledons (averaged ratings of 7.3–9) while inoculation with the remaining two isolates (00-100s and WA30s) resulted in intermediate interactions (5.5. and 5.4, respectively). In contrast, seven of the eight isolates proved to be avirulent on the IL Topas-*LepR2* (average scores 3.1 to 4.8) and only the reference isolate v23.1.3 proved to be truly virulent against *LepR2* (8.2). Variation in resistance response was also clearly shown for *Rlm3*, in which we show a wide range of averaged lesion scores (2.0–6.0 for interactions with *AvrLm3* isolate 00-100s) over 23 variety lines harboring the gene (Tables 2, 3). We speculate that this phenomenon can be attributed to variation in the genomic background of individual varieties, whereby the amplitude of HR is affected, either positively or negatively, by other host loci involved in recognition or signaling during pathogen invasion. The recognition of the *L. maculans* effectors *AvrLm1* (Gout et al., 2006) and *AvrLm2* (Ghanbarnia et al., 2015) by the *R* genes *LepR3* (Larkan et al., 2013) and *Rlm2* (Larkan et al., 2015), respectively, is known to involve at least two additional partner proteins in the membrane-bound signaling complex; SOBIR1 and BAK1 (Larkan et al., 2015; Ma and Borhan, 2015). Other host proteins may also be involved in the signaling complex, based on other plant interactions with apoplastic fungal pathogens (van der Hoorn and Kamoun, 2008; Stotz et al., 2014; Misas-Villamil et al., 2016). Subtle variation in either the expression or sequence of these partner proteins may account for the range of resistance responses observed. Indeed, in each case where we compared an *R* gene present in a largely Westar-derived genomic background; 1065 (*LepR1*), 1135 (*LepR2*), DH12075 (*Rlm3*) and the *Rlm3* variety AC Excel (a selection of Karat x Westar Rakow, 1993), against the Topas DH16516-derived genomic background of the ILs (Topas-*LepR1*, Topas-*LepR2* and Topas-*Rlm3*) we observed significantly stronger HR and much lower variance in IL cotyledon ratings across the test (Tables 2, 3, Figure 3). This would suggest that the Westar genomic background is somewhat deficient in its ability to fully express *R* gene-mediated cotyledon resistance. Another notable discrepancy was the comparatively weak reaction of *Rlm1* in the donor parent line Quinta DH24288 compared to Topas-*Rlm1* or Columbus when challenged with isolate v23.1.3 (*AvrLm1*) or the transgenic isolate 3R11:*AvrLm1* (Table 2), again suggesting that substitution of the genomic background can affect the amplitude of the *R* gene response and hence the ability of the observer to accurately infer the presence or absence of corresponding *Avr* genes in the pathogen or the *R* gene present in the respective host plant.

Additionally, we have also observed what appears to be an effect of heterozygosity on the amplitude of HR. In particular, during the mapping of *Rlm7*, it was very difficult to determine the resistance status of individual BC_1_F_1_ seedlings, with most appearing intermediate to susceptible by 14 dpi. Only after assessment of the resulting BC_1_F_2_ progeny, where individuals harboring homozygous *Rlm7* could be observed, was it possible to confidently assign resistance or susceptibility to each member of the mapping population (data not shown). This suggests a co-dominant phenotype for *Rlm7*. Additional testing for potential co-dominant phenotypic effects is required for blackleg *R* genes, particularly in regards to the accurate assessment of *R* gene content in modern hybrid (heterozygous) varieties.

Our results for the detection of *Rlm3* and *Rlm4* in *B. napus* cultivars conflict with several other reports in the literature. The blackleg *R* gene *Rlm4* was first identified in the varieties Jet Neuf and Quinta V, a single-plant selection of cv. Quinta (Balesdent et al., 2001). However, in determining the *Rlm4* resistance phenotype for Quinta V selection, the authors remarked on the heterogeneous nature of the Quinta variety seed, noting the isolate used to characterize the *Rlm4* interaction showed virulence on the original seed lot obtained, which appeared to segregate for both *Rlm1* and *Rlm4* (Balesdent et al., 2001). As demonstrated in the present study and noted in previous reports (Kutcher et al., 2010; Larkan et al., 2013), the DH line Quinta DH24288, produced at AAFC Saskatoon from a Quinta seed lot that was originally obtained as breeder seed (Mengistu et al., 1991; P. H. Williams, pers. comm), is homozygous for *Rlm1* and *Rlm3*. Genetic characterisation of the A07 blackleg *R* gene locus has a long history. A single dominant *R* gene was originally mapped from the French cultivar Major and termed LEM1 (Ferreira et al., 1995). LEM1 was later shown to induce resistance to the *L. maculans* PG2 isolate PHW1245 carrying the avirulence gene *alm1* (Pongam et al., 1998). LEM1 from Major was later assumed to be *Rlm4*, as were mapped resistance loci LmFr1 from French cv. Crésor (Dion et al., 1995) and LmR1 from the Australian cultivars Shiralee and Maluka (Mayerhofer et al., 1997), based on map location and/or differential pathology tests (Rouxel et al., 2003b; Rimmer, 2006). The Crésor resistance, present in DH12075, a DH line produced from a Crésor × Westar F_1_, was again mapped (as CLmR1) and shown to localize to the same chromosomal position as LmR1 from Shiralee (Mayerhofer et al., 2005). Our tests suggest that all of these lines in fact carry *Rlm3*, not *Rlm4*. This is supported in part by previous work in which cultivars Shiralee, Maluka, Quinta, and Major were all shown to be susceptible to an isolate carrying *AvrLm4-7* (Raman et al., 2012b) and also by the pedigree of the *Rlm3* varieties Quantum and Q2, which contain *L. maculans* resistance derived from Maluka and Shiralee, respectively (Stringam et al., 1995a,b; 1999). However, the *B. napus* variety lines tested here have been selected over several generations for homozygous phenotypes when challenged with *L. maculans*, with several of the selections coming from source seed that was clearly segregating for *R* gene content on first assessment (data not shown). The variety lines used in the testing therefore do not necessarily represent the cultivar as it was upon commercial release, particularly in regards to the homozygosity of the *R* genes carried by the variety, nor do they necessarily represent the variety lines of the same name held by other institutions. Disparities in either *R* gene classification of *B. napus* varieties or pathotyping of *L. maculans* isolates, due to either error or variation in germplasm, only highlights the need for unambiguous, standardized material for pathological studies.

*Brassica napus* is an allotetraploid species, consisting of two highly-homologous diploid genomes known to undergo inter-genomic homoeologous recombination which can result in complex genomic rearrangements (Nicolas et al., 2007; Cai et al., 2014; Mason et al., 2014). Three of the ILs appear to be missing the lower portion of chromosome C06, likely due to the parental lines containing non-compatible A07-C06 genomic arrangements (Osborn et al., 2003). The whole-genome SNP analysis indicated that these lines carry two copies of lower A07; one copy from the *R* gene donor parent and a second copy replacing lower C06, derived from Topas DH16516. This event affects C06 from approximately 12.6 Mb to the end of the lower chromosome arm, with the duplication occurring at approximately 16.85 Mb on A07, around 550 kb below the mapped *Rlm3* and *Rlm4* interval, leaving these genes unaffected. However, the *Rlm1* region of A07, positioned approximately 4 Mb below this point (Figure 1) is represented twice in the Topas-*Rlm1* IL. We infer that Topas-*Rlm1* carries the Quinta DH24288 *Rlm1* allele on A07, while the Topas DH16516 *rlm1* allele is carried on C06. This duplication of lower A07 does not appear to affect the phenotypic expression of the *R* genes in question, with all three ILs producing consistent resistance responses equal to or better than other *B. napus* varieties harboring the same *R* genes. However, this genomic event is something that should be taken into consideration when analyzing these lines in complex, whole-genome molecular studies. Significant homoeologous translocation events were also detected in several of the other *B. napus* varieties surveyed, the most notable resulting in very large null regions detected for lower C01 (22.92 Mb) in Goéland and for upper C02 (33.68 Mb) in Scoop (Supplementary Table 5). In these cases no marker heterozygosity was detected for the corresponding homoeologous A genome regions, suggesting these varieties are affected by non-reciprocal translocations, where the varieties carry two highly-monomorphic copies of the corresponding A01 and A02 chromosomal regions, respectively, with total loss of the affected the C genome regions.

Our hope is that the ILs described here will be widely distributed amongst the *Leptosphaeria* research community and will provide a strong basis for comparison of *Leptosphaeria*—*Brassica* interactions between labs. By introgressing the *R* genes into a common genomic background we have produced highly standardized material for *Leptosphaeria* pathology and related studies of host gene expression, and largely eliminated both negative and positive background variation which could interfere with the unambiguous pathotyping of *L. maculans* isolates and determination of *R* gene- specific defense responses. Work to add to the IL set through the production of new ILs (*Rlm7, Rlm9* etc.) is on-going.

## Author contributions

NL, FY, DL, SR, and MB designed experiments. NL and FY carried out production of ILs and genetic mapping of *R* loci. NL performed all final pathological and genomic characterisations of lines. NL and MB drafted manuscript. All authors read and approved final manuscript with the exception of SR (deceased).

## Funding

This work was funded by the Western Grains Development Fund, the AAFC-Industry Blackleg Consortium II (members include Agriculture Victoria Services Pty Ltd on behalf of the State of Victoria, Australia, Bayer, Crop Production Services (Canada), Dow AgroSciences, Lantmännen and Monsanto Canada) and the AAFC Canadian Agri-Science Cluster.

### Conflict of interest statement

The authors declare that the research was conducted in the absence of any commercial or financial relationships that could be construed as a potential conflict of interest.
