# Supplementary material for: Single R Gene Introgression Lines for Accurate Dissection of the Brassica - Leptosphaeria Pathosystem
Source: Front Plant Sci. 2016 Nov 28;7:1771. doi: 10.3389/fpls.2016.01771 (PMC5124708; doi:10.3389/fpls.2016.01771)
Supplement: Supplementary file 1 [file DataSheet1.PDF]

Supplementary Table 1. Parental *B. napus* varieties used for generation of Introgression Lines.

IL	*R* parent	F_1 S parent	BC_1 S parent	BC_2 to BC_5 S parent
Topas-*Rlm1*	Quinta DH24288	Topas variety	Topas variety	Topas DH16516
Topas-*Rlm2*	Glacier DH24287	Topas variety	Topas variety	Topas DH16516
Topas-*Rlm3*	Glacier DH24287	Topas variety	Topas variety	Topas DH16516
Topas-*Rlm4*	Scoop	Topas DH16516	Topas DH16516	Topas DH16516
Topas-*LepR1*	1065	Topas DH16516	Topas DH16516	Topas DH16516
Topas-*LepR2*	1135	Topas DH16516	Topas DH16516	Topas DH16516
Topas-*LepR3*	Surpass 400	Topas DH16516	Topas DH16516	Topas DH16516

Supplementary Table 2. *L. maculans* Avr gene cloning primers for transgenic isolates.

Primer Name	Sequence
GW-AvrLm3G-F	GGGGACAAGTTTGTACAAAAAAGCAGGCTTCTTATTTCCTGCAAGACGCTAGTAGCGC
GW-AvrLm3G-R	GGGGACCACTTTGTACAAGAAAGCTGGGTCCCTGTTAAATGCCTGCTGTTTTAGCC
GW-AvrLm6G-F	GGGGACAAGTTTGTACAAAAAAGCAGGCTTCGGCAAATAATAAAGCCGCTAGATTCAACGTTAGA
GW-AvrLm6G-R	GGGGACCACTTTGTACAAGAAAGCTGGGTCAGCACTACGTGCACGCTTAGATACGAATCAA

Supplementary Table 3. Single-nucleotide polymorphism (SNP) marker calls. a) Comparison of SNP polymorphism between Topas DH16516 (DHT), *R* gene donor parents and ILs, b) SNP polymorphism between Topas DH16516 and other *B. napus* control lines.

a)

		Parental and Introgression Lines														
		**Topas DH16516 Mean**	**Topas Mean**	Quinta DH24288 (*Rlm1*, *Rlm3*)	**T-*Rlm1* Mean**	Glacier DH24287 (*Rlm2*, *Rlm3*)	**T-*Rlm2* Mean**	**T-*Rlm3* Mean**	Scoop 2-1 (*Rlm4*)	**T-*Rlm4* Mean**	1065 (*LepR1*)	**T-*LepR1* Mean**	1135 (*LepR2*)	**T-*LepR2* Mean**	Surpass 400 (*LepR3*, *RlmS*)	**T-*LepR3* Mean**
DHT	Homo	**41302**	**34546**	19805	**39882**	19688	**40871**	**38696**	24290	**39735**	30851	**40939**	30035	**40630**	27579	**40675**
Alleles	Hetero	**1243**	**745**	507	**969**	493	**1150**	**875**	478	**978**	661	**1195**	629	**1076**	556	**1068**
	null	**631**	**441**	180	**613**	169	**615**	**563**	238	**597**	329	**621**	296	**591**	256	**626**
Non-DHT	Homo	**0**	**4981**	19178	**588**	19543	**339**	**1891**	12862	**734**	9148	**225**	9014	**506**	12099	**275**
Alleles	Hetero	**0**	**1909**	2569	**351**	2571	**103**	**578**	2130	**475**	1434	**139**	2236	**79**	1761	**87**
	null	**36**	**589**	972	**808**	747	**134**	**609**	3213	**692**	788	**93**	1001	**328**	960	**480**
	Sum	**43211**	**43211**	43211	**43211**	43211	**43211**	**43211**	43211	**43211**	43211	**43211**	43211	**43211**	43211	**43211**
	%DHT	**0.999**	**0.827**	0.474	**0.96**	0.471	**0.987**	**0.929**	0.579	**0.956**	0.737	**0.989**	0.716	**0.979**	0.657	**0.981**

b)

		Other *B. napus* Controls											
		Westar No1	Columbus	Bristol	DH12075	Quantum	AG-Castle	Jet Neuf	Falcon	Roxet	Darmor	Goeland	NLA 8-2
DHT	Homo	31348	22267	19726	27824	26230	24867	20221	19787	19489	21242	19769	41281
Alleles	Hetero	704	543	474	620	658	565	495	468	477	479	438	1242
	null	330	227	170	326	302	218	162	145	148	159	165	631
Non-DHT	Homo	8374	11132	19127	11989	12607	14122	18695	19161	19489	17831	17443	0
Alleles	Hetero	1751	7123	2733	1763	2228	2116	2543	2757	2717	2682	2538	2
	null	704	1919	981	689	1186	1323	1095	893	891	818	2858	55
	Sum	43211	43211	43211	43211	43211	43211	43211	43211	43211	43211	43211	43211
	%DHT	0.749	0.533	0.471	0.666	0.629	0.594	0.483	0.472	0.465	0.506	0.471	0.999

Supplementary Table 4. Significant donor parent introgressions and null regions detected in each IL. Introgression corresponding to mapped position of each *R* gene highlighted by box. Flanking marker positons given as Darmor-*bzh* reference genome chromosomal coordinates. Abbreviations for introgression donor parent lines; Topas var. = Topas variety line, DHQ = Quinta DH24288, DHG = Glacier DH24287, S400 = Surpass 400.

IL	Chrom.	Flanking Markers		Donor	Status	Size
**Topas-*Rlm1***	A03	1,649,707	1,963,409	Topas var.	Segregating	313,702
	A06	2,867,926	2,943,692	Topas var./DHQ	Segregating	75,766
	A06	21,623,427	21,644,014	Topas var./DHQ	Fixed	20,587
	A06	22,298,045	22,640,203	Topas var.	Fixed	342,158
	A07	16,644,453	18,986,802	DHQ	Fixed/hetero	2,342,349
	A07	19,039,999	20,124,052	Topas var.	Fixed/hetero	1,084,053
	A07	20,708,529	21,632,232	DHQ	Fixed/hetero	923,703
	A08	212,846	9,589,505	Topas var.	Fixed	9,376,659
	C01	17,763	4,491,212	null	Fixed	4,473,449
	C06	24,587,541	37,194,889	null	Fixed	12,607,348
**Topas-*Rlm2***	A01	1,044,861	1,730,502	Topas var.	Fixed	685,641
	A04	15,545,720	15,748,927	Topas var.	Fixed	203,207
	A09	8,414,770	8,740,005	Topas var.	Fixed	325,235
	A09	19,445,863	23,062,383	Topas var.	Fixed	3,616,520
	A10	13,447,460	15,638,228	DHG	Fixed	2,190,768
	C04	2,958,090	4,500,056	Topas var.	Segregating	1,541,966
	C04	34,315,607	36,165,667	Topas var.	Segregating	1,850,060

	C04	37,701,744	40,370,658	Topas var.	Segregating	2,668,914
	C04	45,535,992	45,613,624	Topas var.	Fixed	77,632
**Topas-*Rlm3***	A02	21,945,355	22,899,433	DHG	Fixed	954,078
	A02	24,065,091	24,551,863	DHG	Segregating	486,772
	A03	4,423,261	20,381,953	Topas var.	Fixed	15,958,692
	A05	22,090,877	23,024,268	Topas var.	Fixed	933,391
	A06	3,565,417	4,596,998	DHG	Fixed	1,031,581
	A07	18,576	2,704,430	DHG	Fixed	2,685,854
	A07	8,360,439	8,546,733	DHG	Fixed	186,294
	A07	15,278,845	15,862,878	Topas var.	Fixed	584,033
	A07	15,862,878	16,936,576	DHG	Fixed	1,073,698
	A07	16,936,576	22,229,454	DHG	Fixed/hetero	5,292,878
	A07	22,229,454	23,356,664	DHG	Fixed	1,127,210
	A07	23,356,664	23,947,641	DHG	Segr./hetero	590,977
	A09	30,998,020	33,861,419	DHG	Fixed	2,863,399
	C01	7,148,849	9,998,448	Topas var.	Fixed	2,849,599
	C01	10,893,262	11,329,696	Topas var.	Fixed	436,434
	C04	1,513	47,948	Topas var./DHG	Fixed	46,435
	C06	24,587,541	37,194,889	null	Fixed	12,607,348
	C07	32,175,473	36,077,540	Topas var.	Fixed	3,902,067
	C07	36,077,540	39,906,379	Topas var.	Segregating	3,828,839
	C07	39,906,379	42,935,865	Topas var.	Fixed	3,029,486
	C07	43,096,577	43,717,958	DHG	Fixed	621,381
	C08	722,301	1,383,439	Topas var.	Fixed	661,138
	C08	5,934,073	25,733,332	DHG	Fixed	19,799,259
	C08	32,753,010	33,198,932	Topas var.	Fixed	445,922
	C08	34,731,449	38,386,401	DHG	Fixed	3,654,952
**Topas-*Rlm4***	A01	2,359,439	2,449,284	Scoop	Fixed	89,845
	A01	4,049,901	10,421,790	Scoop	Segregating	6,371,889

	A01	2,700,421	4,049,901	Scoop	Fixed	1,349,480
	A04	18,387,031	19,140,178	Scoop	Fixed	753,147
	A07	10,559,266	16,792,643	Scoop	Fixed	6,233,377
	A07	16,792,643	23,093,446	Scoop	Fixed	6,300,803
	A08	9,933,069	9,978,124	Scoop	Fixed	45,055
	A09	6,875,368	26,387,619	Scoop	Segregating	19,512,251
	A09	28,461,969	30,119,108	Scoop	Segregating	1,657,139
	C03	2,433	3,217,123	Scoop (null)	Segregating	3,214,690
	C06	24,587,541	37,194,889	null	Segregating	12,607,348
**Topas-*LepR1***	A02	10,022,968	20,435,480	1065	Fixed	10,412,512
	A05	1,397,395	19,824,879	1065	Segregating	18,427,484
	C02	24,017,753	24,033,857	1065	Fixed/hetero	16,104
	C04	2,580,653	3,581,178	1065	Segregating	1,000,525
	C07	27,924,277	29,852,608	1065	Segregating	1,928,331
**Topas-*LepR2***	A10	203,782	14,156,599	1135	Fixed	13,952,817
	C05	1,305,483	1,654,223	1135	Fixed	348,740
	C08	17,676,780	18,814,963	1135	Segregating	1,138,183
	C09	34,336,178	35,821,709	1135	Fixed	1,485,531
**Topas-*LepR3***	A01	29,929	6,397,251	S400	Fixed	6,367,322
	A10	13,322,911	16,388,702	S400	Fixed	3,065,791
	A10	16,391,071	16,640,143	S400	Segregating	249,072
	C03	6,666,360	7,680,817	S400	Segregating	1,014,457

Supplementary Table 5. Significant null regions detected in other *B. napus* variety lines. Coorinates and size of regions given as bp position as per Darmor-*bzh* reference genome (Chalhoub et al., 2014).

Variety	Chrom.	Coordinates		Size
		Start	End	
Goeland	C01	15,086,844	38,007,459	22,920,615
Quantum	C02	8,254	8,885,685	8,877,431
Scoop	C02	8,254	33,684,876	33,676,622
1065	C03	2,433	2,852,351	2,849,918
1135	C03	2,433	2,852,351	2,849,918
Scoop	C03	2,433	2,852,351	2,849,918
Westar	C03	2,433	2,852,351	2,849,918
Quinta	C03	2,433	3,325,140	3,322,707
Scoop	C07	40,686,921	44,707,512	4,020,591
Jet Neuf	C08	34,919,920	38,386,401	3,466,481

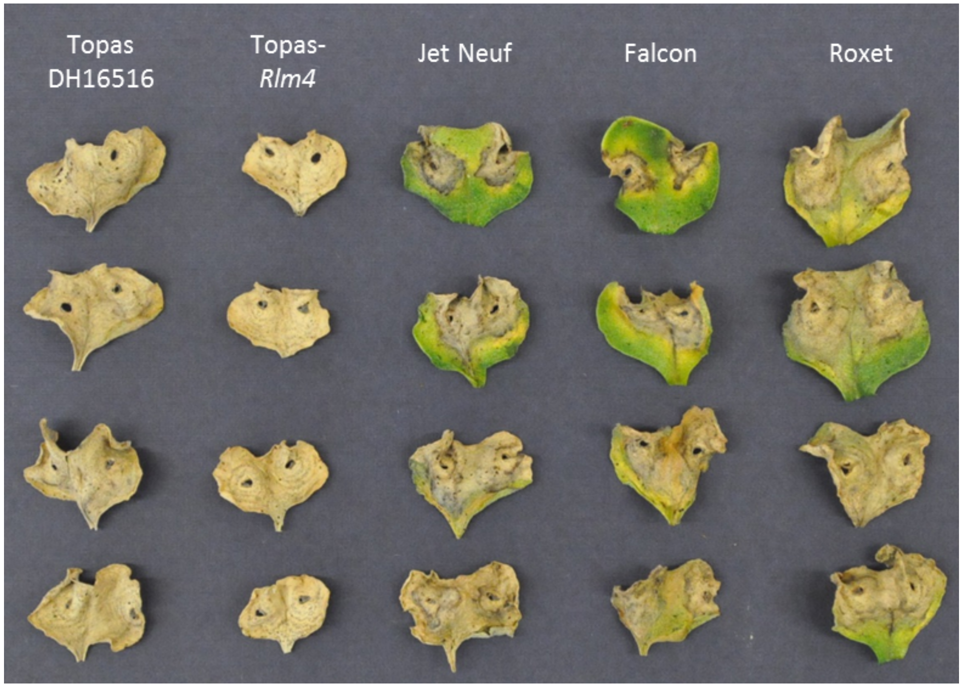

Supplementary Figure 1. Restriction of cotyledon infections on compatible varieties (B14-13s). A selection of cotyledons showing the range of responses observed during testing. Infections shown at 14 dpi.
